# “Resilience in crop reproduction for food security: introducing RECROP COST action”

**DOI:** 10.1007/s00497-024-00497-8

**Published:** 2024-02-14

**Authors:** Sotirios Fragkostefanakis, Michal Lieberman-Lazarovich, David Honys

**Affiliations:** 1 Institute for Molecular Biosciences, Molecular Cell Biology of Plants, Goethe University Frankfurt, Frankfurt, Germany; 2 Volcani Center, Plant Sciences Institute, Agricultural Research Organization, Rishon LeZion, Israel; 3 Laboratory of Pollen Biology, Institute of Experimental Botany of the Czech Academy of Sciences, Prague, Czech Republic; 4 Department of Experimental Plant Biology, Faculty of Science, Charles University, Prague, Czech Republic

## Summary

In response to the escalating threat of climate change and its detrimental impact on plants' productivity and thus on global food security, a new Action called RECROP (Reproductive Enhancement of CROP resilience to extreme climate CA22157) has emerged, supported by the European Cooperation in Science and Technology (COST). Comprising an exceptional consortium of agronomists, physiologists, geneticists, biologists and bioinformaticians from academia, private sector, public institutions and other relevant organisations. RECROP sets out to support and transform current efforts for improving crop resilience through innovative and holistic approaches. This collaborative effort aims to unlock the secrets of crop sensitivity to environmental stresses during plant reproduction and design solutions to enhance crop yields in a sustainable manner.

## A dire need for resilient crops

The alarming rise in extreme weather events poses a significant threat to the world's major crops (Rivero et al. 2022). Among them, grain and fruit crops, which form the bedrock of the human diet, are particularly vulnerable due to their sensitivity to abiotic stresses and their combinations, mainly due to the sensitivity of reproductive organs. As temperatures soar, droughts intensify, and floods become more frequent, the critical process of sexual reproduction in crops faces unprecedented challenges (Chaturvedi et al. 2021). With global food security at stake, the urgency to develop crop varieties with enhanced reproductive stress resilience cannot be ignored.

## A holistic vision to a global problem

To address this pressing issue, RECROP brings together a diverse group of experts from various scientific disciplines. Under the RECROP platform we aim to understand the genetic, molecular, and physiological attributes to crop sensitivity during reproduction and devise effective strategies to boost yield amidst the evolving climate crisis. Through the integration of interdisciplinary approaches, RECROP aims to generate comprehensive models and cutting-edge solutions.

## Unlocking the genetic code of resilience

RECROP focuses on defining standards for experimental setups, data collection, analysis, and interpretation of results. Its comprehensive approach will encompass both wet laboratories and in silico methodologies, leading to a deeper understanding of crop responses to high temperatures, droughts, and floods. By identifying specific genes and pathways as targets for crop improvement, RECROP aims to pave the way for resilient crop varieties that can withstand adverse climate conditions. The validation of crop models and hypothesis testing will facilitate benchmarking of pipelines and workflows, fostering collaborative research and innovation. By sharing knowledge, infrastructure, datasets, genetic and computational tools, RECROP seeks to create an inclusive and accessible research environment that nurtures a collective effort towards increasing food security for a sustainable future.

## Fostering Open Science and Collaboration

At the heart of RECROP lies the commitment to foster Open Science and promote collaboration among scientists from diverse backgrounds and different career stages. RECROP aims to create a knowledge-sharing platform to enable the seamless collaboration of researchers. By encouraging open access to data, methodologies, and findings, RECROP seeks to accelerate scientific progress and catalyse innovative solutions for crop reproduction resilience in the face of changing and challenging climate. RECROP places immense emphasis on empowering early stage career researchers and committed students. By promoting networking, training, and engagement in strategic collaborations, RECROP aims to foster a new generation of scientists driven to tackle the complex challenges posed by climate change. In addition, RECROP will offer insights into the current status and future projections of crop yields to policy makers and the general public, to set the ground and support the development of evidence-based policies in biotechnology, technology, and agricultural sectors.

## A vision for a global food security

As the world grapples with the *sui generis* unparalleled impacts of climate change, RECROP aspires to reshape the landscape of crop resilience towards ensuring food security. This ambitious undertaking not only promises to revolutionize agricultural practices but also holds the potential to support the well-being of humanity in the face of a changing climate. If you are a passionate scientist committed to pioneering advancements in crop reproduction resilience and securing global food security in this era of climate change, we invite you to be part of the RECROP initiative. Join RECROP in our quest to empower crops, enhance their reproductive potential, and cultivate a sustainable and resilient tomorrow. For more information on our Action, updates and activities visit www.recrop-cost.com. To become RECROP member visit www.cost.eu/actions/CA22157/. RECROP started officially in October 2023 and will run for 4 years.
